# Risk perception and precautionary health behavior toward COVID-19 among health professionals working in selected public university hospitals in Ethiopia

**DOI:** 10.1371/journal.pone.0241101

**Published:** 2020-10-29

**Authors:** Shimelis Girma, Liyew Agenagnew, Girma Beressa, Yonas Tesfaye, Arefayne Alenko

**Affiliations:** 1 Department of Psychiatry, Institute of Health, Jimma University, Jimma, Ethiopia; 2 Department of Public Health, School of Health Sciences, Madda Walabu University, Goba, Ethiopia; University of New South Wales, AUSTRALIA

## Abstract

**Objective:**

This study was designed to determine risk perception and precautionary health behavior toward coronavirus disease (COVID-19) among health professionals working in selected public university hospitals of Ethiopia.

**Methods:**

A web-based cross-sectional survey was used with health professionals working in selected public university hospitals of Ethiopia. A structured survey questionnaire consisted of socio-demographic, risk perception, and behavioral response items were used. The survey questions were designed on Google form. All academic and clinical staff invited to participate in the online survey, which was carried out from May 1–14, 2020. Data analysis was done using the Statistical Package for Social Sciences version 24. Descriptive statistics computed and the result is presented by tables and figures.

**Results:**

A total of 273 health professionals participated in this study. The mean (± SD) age of participants was 31.03 ± 5.11. Study participants’ overall mean score of perceived risk was 23.59 ± 4.75. The study participants’ mean score of perceived vulnerability (4.01 ± 1.17) was higher than the human immunodeficiency virus, common cold, malaria, and tuberculosis. Regarding precautionary health behavior, the lowest mean score is for wearing gloves 1.82 ± 1.15.

**Conclusion:**

Participants mean score of perceived vulnerability of coronavirus disease was higher than some of the prevalent infectious disease in the area. Almost all participants applied recommended protective measures to the acceptable level, except for wearing mask and gloves. Therefore, there is a need to further intensification of more effective ways to support health professionals’ adherence to major precautionary measure is important.

## Introduction

The recently emerging novel coronavirus disease, COVID-19, is causing massive death, threatens the wellbeing of the global society, and causing large economic loss since it is declared a pandemic by the World Health Organization (WHO) [1]. The virus is highly contagious, which causes difficulty in controlling the outbreak [2]. The only proven preventive option is to implement the recommended public health measures, which include the use of personal protective measures such as the use of face masks, wearing of gloves, frequent hand washing, restriction of movement to the more affected areas, maintaining social/physical distance, avoiding touching the nose, mouth, and eyes, were found to be the only proven preventive option to halter the pandemic [3]. Health care workers, especially those whose jobs allow more contact with patients infected with COVID-19, are at risk of adverse health consequences [4, 5]. They are working with inadequate personal protective equipment (PPE), and thus many have died and thousands have tested positive [6].

To adhere to the recommended protective measures, individuals risk perception is one of the major factors that need to be considered [7]. The effectiveness of outbreak control will mainly depend on the behavioral response of the society and adherence level to the recommended precautionary measures [8]. Poor understanding and risk perception of the disease among health care workers (HCWs) may result in delayed recognition and treatment, resulting in the rapid spread of the infection. A significant number of health care workers have inadequate knowledge and poor perceptions of COVID-19. This is evidenced by a cross-sectional, web-based study among HCWs about COVID-19 during the first week of March 2020. According to this study, 61% and 63.6% of HCWs had poor knowledge about COVID-19 transmission and symptom onset, respectively. Besides, a significant number of HCWs (22%) showed negative perceptions of COVID-19 [9].

Since May 20, 2020, there are about 365 COVID-19 confirmed cases and 5 deaths reported in Ethiopia [10]. In this time, the disease outbreak is affecting the big cities and towns in the gateways. As the country is in the early stage of infectious disease, the chance of limiting the spread is more feasible during this stage. Thus, the online survey was intended to assess health professionals’ risk perception and their precautionary behavioral responses. The finding is essential in haltering the outbreak, and it is important in recommending concerned bodies to implement necessary intervention strategies that safeguard the lives of health care providers.

## Methods and materials

### Study design and setting

A self-administered online cross-sectional survey was designed on Google form and administered from May 1–14, 2020. The study was conducted on health professionals working in selected public university hospitals in South, Southwest, and Western Ethiopia and it is a part of an online survey titled “COVID-19 pandemic: psychological impact, perceived risk and preparedness among health care providers working in public university hospitals in Ethiopia: web-based survey.” The university hospitals included in the study are namely: Jimma Medical Center (JMC), Mizan Tepi University Hospital, Wollega University Hospital, Hawassa University Hospital, Wolayta Sodo University Hospital, and Arba Minch University Hospital. All University hospitals were engaged in delivering curative treatment, preventive, and rehabilitative services for millions of people in the south, southwest, and western Ethiopia. For the current COVID-19 pandemic, each university hospital was made ready for testing, treatment, and quarantine center.

### Population

All teaching and clinical staff working in the selected public university hospital of Ethiopia was considered a source population. A survey link was sent to 864, 521, 343, 271, 123, and 223 staffs of Jimma University, Arba Minch University, Hawassa University, Mizan Tepi University, Wolayta University, and Wollega University staffs, respectively. A total of 273 health professionals took part in the study. Those who were on work leave were not invited to take part in the study.

### Sample size and sampling techniques

The minimum number of the sample size required for this study was determined by using the formula to estimate a single population proportion. The minimum sample size determination formula used is: $(n)=\frac{{\left(\frac{Z\alpha }{2}\right)}^{2}P(1-P)}{d^{2}}$, where *n* denotes the minimum sample size, Z α/2 is the reliability coefficient of the standard error at a 5% level of significance = 1.96, and p is the proportion of health care providers who had poor perception about COVID-19 = 22% [9]. Accordingly, the study participants were selected consecutively.

### Data collection procedure and instrument

Data were collected by an electronically administered questionnaire. All staff was invited to participate in the online survey. Official university e-mail addresses, personal e-mail addresses, and social media networking sites including Facebook and Telegram were used to share the survey link to the staff. The information regarding the purpose of the study, informed consent, and confidentiality of the data were explained for the study subjects in the introduction part of the online survey. The survey questions were adapted from WHO resources and other related studies which were previously used for infectious outbreak like a severe acute respiratory syndrome (SARS) [8, 11, 12]. The questionnaire was delivered electronically by the English language to get a rapid response to individuals’ risk perception and behavioral response. The survey instrument included the socio-demographic characteristics of the participants, participants’ risk perception which was assessed by a 10-item perceived risk scale. The scale is rated on a 5-point Likert scale and assesses perceived threat level, perceived risk of infection, perceived severity of the infection and perceived vulnerability to infection, and respondents’ self-efficacy. The behavioral responses of respondents was assessed by asking respondents questions like avoiding sneezing/couching, avoiding touching the face, nose, and eyes, avoiding large gatherings and public places, avoiding traveling to the affected areas, using alcohol-based disinfectants, and wearing masks and gloves. The scale allows to rate the response of the respondent in the ordered of 1—least likely, to 5—most likely.

### Data processing and analysis

The data were checked, coded, and exported to Statistical Package for Social Sciences (SPSS) version 24 statistical software for analysis. Descriptive statistics were computed and presented with a frequency table indicating the mean value and standard deviation with a 95% confidence interval.

### Ethical consideration

Ethical approval obtained from the Institute of Ethical Review Board of Jimma University with letter reference number IRB000212/2020. Informed consent was obtained from all study participants. Information regarding the purpose of the study, voluntary nature of participation, and risk imposed due to involvement presented in the information section of the survey. The survey questionnaire designed in a way the study participants only directed to the survey questions following a respondent click on a response button “Agree to participate” after reading the consent information. To ensure anonymity, information identifying the participants was not included in the survey. Furthermore, the data files stored on a password-protected computer.

## Results

### Characteristics of the study participants

A total of 273 health professionals participated in this study. One-thirds 80 (29.3%) of study participants is from JMC. The mean (± SD) age of participants was 31.03 ± 5.11 years, and range from 23 to 48 years. Two-thirds 177 (64.8%) and one-fourths 71 (26%) of the study participants were engaged in academic service and medical doctors by profession, respectively. Almost all study participants are living in an urban area. Regarding educational attainment and sex of respondents, 61.5% and 89% of participants attended the Master’s Degree (MSc) and male by sex, respectively (**Table 1**).

**Table 1:** Socio-demographic characteristics of health professionals working in public university hospitals of Ethiopia, May 2020 (n = 273).

Variables	Variable categories	Frequency	Percentage (%)
Sex	Male	243	89
	Female	30	11
Age category	15–24	18	6.6
	25–34	201	73.6
	≥ 35	54	19.8
Educational qualification	Bachelor Degree	96	35.2
	Masters Degree	168	61.5
	PhD	9	3.3
Type of profession	Nursing/Midwifery	121	44.3
	Medical doctors	71	26.0
	Anaesthesia/psychiatry/Ophthalmology	46	16.8
	Public health	35	12.8
Professional engagement	Academics	177	64.8
	Clinical	81	29.7
	Both academics and clinical	15	5.5
Residence	Urban	267	97.8
	Rural	6	2.2
Participants’ working institution	Jimma Medical Center	80	29.3
	Arba Minch University	56	20.5
	Hawassa University	55	20.1
	Wolayta University	28	10.3
	Mizan Tepi University	27	9.9
	Wollega University	27	9.9

### COVID-19 risk perception, vulnerability, self-efficacy, and severity

Regarding risk perception, the overall mean score was 23.59 ± 4.75, and the highest mean score is for perceived getting an infection (3.67 ± 1.04) and the risk of having serious infection because of COVID-19 (3.48 ± 1.13), respectively (**Table 2**). The mean score of perceived vulnerability to COVID-19 (4.01±1.17) of study participants was higher than human immunodeficiency virus (HIV), common cold, malaria, and tuberculosis. Regarding the mean score of perceived severity, COVID-19 rated second next to HIV/AIDS. Study participants were asked a question “*How do you rate perceived ability to avoid infection with COVID-19*?” to rate their self-efficacy on a 5-point scale. Accordingly, the mean score of the participants was found to be (4.01±1.17) (**Table 3**).

**Table 2:** Perceived risk item mean score of health professionals working in public university hospitals of Ethiopia, May 2020 (n = 273).

Items (scored by 5-point Likert scale)	Mean	SD
How do you rate perceive risk of getting infected by COVID-19?	3.67	1.04
How do you rate perceive risk of having serious illness by COVID-19?	3.48	1.13
How do you rate perceive risk of death by COVID-19?	2.8	1.02
How do you rate perceived effect that Corona virus pandemic poses difficulty in your life?	3.02	1.21
How do you rate that Ethiopian are to contract the virus?	3.29	1.15
How do you rate that that people in your present location are to contract the Coronavirus?	3.33	1.05
How do you rate your worry that your family members or friend might be infected by Corona Virus?	2.79	0.92

**Table 3:** Mean score of perceived severity and vulnerability of health professionals working in public university hospitals of Ethiopia, May 2020 (n = 273).

Disease	Severity		Vulnerability	
	Mean	SD	Mean	SD
COVID-19	3.63	1.18	4.01	1.17
HIV/AIDS	3.81	1.23	3.61	1.43
Common Cold	3.33	1.38	3.87	1.2
Malaria	2.89	1.2	3.32	1.56
Tuberculosis	3.42	1.39	3.64	1.45

### Precautionary health behavioral practice

**Fig 1:**
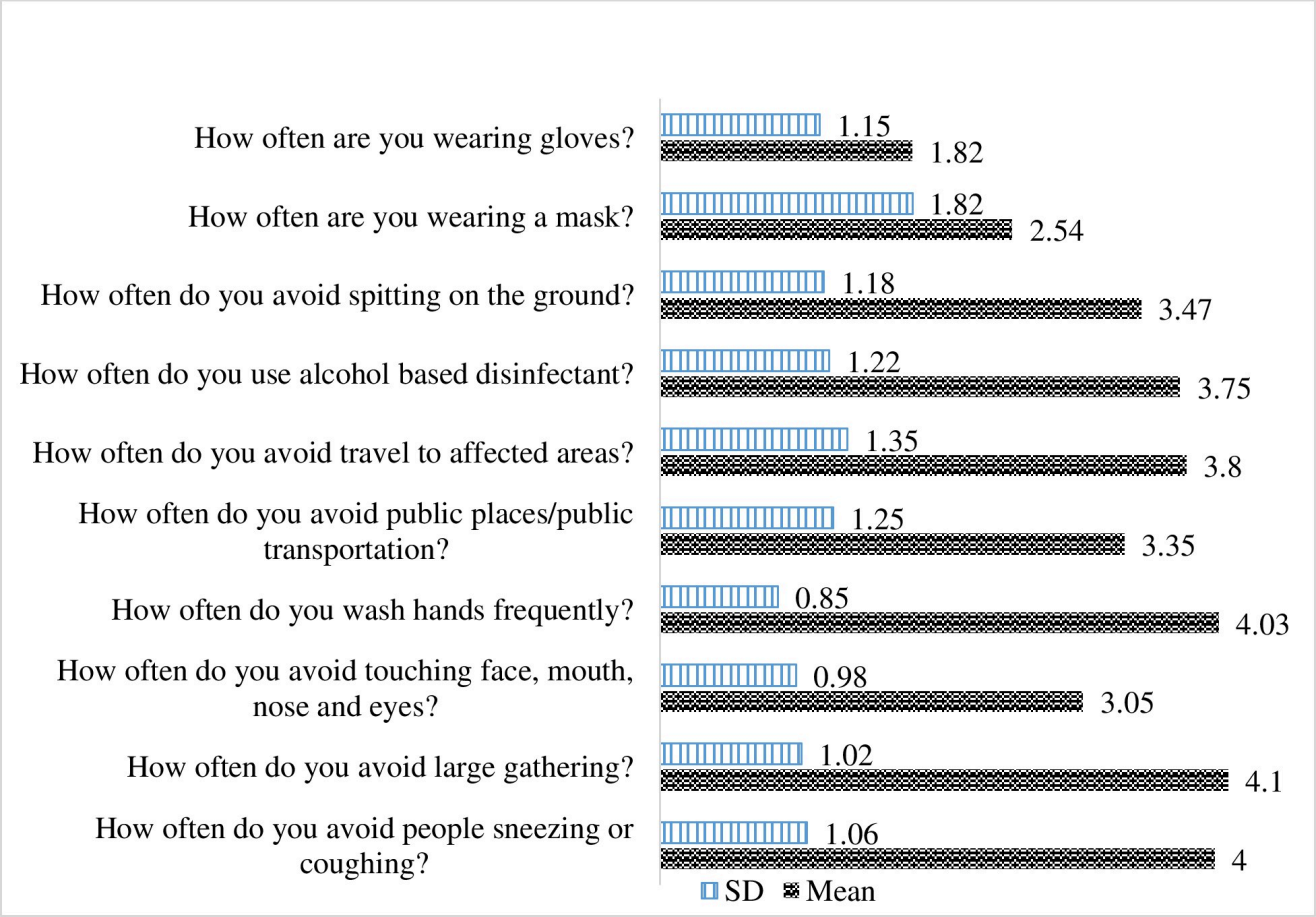
Mean score of precautionary health behavioral practice by health professionals working in selected public university hospitals of Ethiopia, May 2020 (n = 273).

The precautionary behavioral response of study participants was assessed using a 10-item questionnaire that was scored on a 5-point (1–5) Likert scale. The lowest mean scores were wearing gloves (1.82 ± 1.15) followed by wearing a mask (2.54 ± 1.82). Avoiding while people sneeze or cough had the highest mean score (4.0 ± 1.06) (**Fig 1**).

## Discussion

Evidence regarding health professionals perceived risk, severity, vulnerability, and self-efficacy of COVID-19 was generated. The findings indicated that the mean score of perceived vulnerability to COVID-19 is higher than other diseases, including HIV, tuberculosis (TBC), malaria, and the common cold. However, the mean score of perceived severity is second to HIV. Regarding the practice of recommended health behavior, almost all recommended precautionary measures are applied to the highest level except for wearing masks and gloves. The final multiple linear regression analysis indicated a positive association between overall risk perception score, perceived vulnerability, and self-efficacy with a precautionary health behavioral practice.

The overall risk perceptions mean score in this study was found to be 23.59 ± 4.75 (summative score of a 5-point score for the 7-risk perception items). In comparison with this finding, a lower level of risk perception of a severe acute respiratory syndrome (SARS) was reported in Scandinavian countries [13–15]. A possible explanation might be the effect of media and massive public mobilization in Ethiopia. The explanation given for the lower risk perception in Scandinavia was that the media tended to report more risks of abroad and given less attention to the risk inside the country [14, 15]. In this study, overall risk perception was found to be positively associated with health behavioral response. A unit increase in overall risk perception increased the precautionary health behavioral practice by 0.45. Similar studies have shown that the higher the perceived risk, the more people are applying protective measures [16, 17].

The findings of the current study found out that the study participants scored the highest mean score of perceived vulnerability to COVID-19 as compared to the other diseases. Similarly, a unit increase in perceived vulnerability increased the health behavioral response by 1.21. This finding is in line with a study done in Iran. The Iranian study reported the highest perceived threat level towards the new pandemic and a positive and significant correlation between health behavioral response and perceived susceptibility, severity, and self-efficacy [18]. A study that was done during the influenza A (H1N1) outbreak also reported a higher vulnerability towards COVID-19 [19]. This might be because of the realistic awareness of health professionals related to the mode of transmission of the virus, which made the professionals consider the virus more threatening than any other disease. Aside from this, uncertainty in the scientific understanding of the coronavirus might also contribute to the figure. Furthermore, the geographical location of the country has an impact on the perception of professionals, as the disease is unfamiliar in Ethiopia it might be perceived as more severe. A higher level of severity perception of SARS was reported in Europe [11].

Regarding self-efficacy, the mean score of respondent was 2.71 ± 1.04. About 50% of study participants strongly agreed that they can able to control the infection. It is only 5.5% of participants reported that they did not feel that they could control the illness. This is higher than the perceived self-efficacy of SARS for most European countries and lower than Asian countries [11].

Participants’ implementation of the recommended precautionary measures in the current study is to the acceptable level except for wearing masks and gloves. This might be due to the study subjects were well educated, which helped them to better exercise protective behavior, and the higher perceived vulnerability level of the illness. This finding is supported by other studies [12]. Regarding wearing masks and gloves, the mean scores in the current study were 2.54 and 1.82, respectively. The practice is less frequent, despite these is being a key protective measure recommended by the WHO [12]. The lower levels of usage declared in our study may be attributable to the poor habit of mask and glove-wearing, a perception that they interfere with performing tasks, lack of time, and shortage of PPE in the health facilities.

### Strength and limitation

The main strength of this study is that researchers used a Likert scale to measure the outcome and explanatory variables, which is much better to measure behavior than the dichotomized response. The limitation of this study was that the measurement tools used relied on participants’ self-reported data, which were prone to recall bias and moreover, as the data collection tools is prepared in English language and this might affected the quality of the data. Consecutive sampling used in this study and the data collection method allows non-representation and over-report the precautionary behavioral practice, respectively. Furthermore, the population in the current survey mostly consisted of higher degree health professionals this may over-estimate the outcomes of the study.

## Conclusions

Participants mean score of perceived vulnerability of coronavirus disease was higher than some of the prevalent infectious disease in the area. Most participants applied recommended protective measures to the acceptable level, except for wearing masks and gloves. There is a need to further intensification of more effective ways of awareness and behavioral change in order to support adherence to major health precautionary measures is important. The health facilities need to in place a safety protocol that encourages and enforces the protective behavioral response and continues monitoring to the adherence protocol need to be followed by the monitory team. Furthermore, ways to positively reinforce the professionals need to be exercised by the facility administrators.

## Supporting information

S1 Questionnaire (DOCX)

S1 Dataset (XLS)

## List of abbreviations

COVID-19: Corona virus disease
HIV: Human Immunodeficiency virus
HCW: Health care worker
MSc: Master’s degree
PPE: personal protective equipment
SD: Standard deviation
SPSS: Statistical Package for Social Sciences
SARS: Severe Acute Respirator Syndrome
TBC: Tuberculosis
WHO: World Health Organization
