# Supplementary material for: Risk perception and precautionary health behavior toward COVID-19 among health professionals working in selected public university hospitals in Ethiopia
Source: PLoS One. 2020 Oct 29;15(10):e0241101. doi: 10.1371/journal.pone.0241101 (PMC7595387; doi:10.1371/journal.pone.0241101)
Supplement: S1 Questionnaire — (DOCX) [file pone.0241101.s001.docx]

**Annex III: Questionnaire**

**Dear Sir/madam,**

The COVID-19 pandemic is the defining global health crisis and the greatest challenge we have faced. The prevention of the crisis involves the participation of entire community. Furthermore, the outbreak is stressful for people around the world since it is declared as pandemic by world health organization (WHO). Following the wide spread of COVID-19 in Africa region and Ethiopia, concern have been raised on safeguarding the health professionals as they are highly vulnerable to the outbreak. Unprecedented spread of the infection largely affected the health professionals and generated shortage.

**Purpose of the study**: This study is designed to assess the risk perception, knowledge and precautionary behavioral response of clinicians and academicians working at different Ethiopian University hospitals towards the COVID-19. The result will be used as a guide for the public health policymakers

**Who approved the study:** The study is approved by Institutional Review Board of Jimma University with letter reference number with letter reference number IRB000212/2020.

**By clicking the below link, you agree to participate voluntarily in this survey and given your consent to use your anonymous data for research.**

You may exit the survey at any stage, and no compensation will be provided.

**Contact address for any question and inquiry:**

[shimelisgirma@gmail.com](mailto:shimelisgirma@gmail.com) or [liyew2003@gmail.com](mailto:liyew2003@gmail.com) or [gberessa@gmail.com](mailto:gberessa@gmail.com) or [arefeaynealenko@gmail.com](mailto:arefeaynealenko@gmail.com) or [yonastesfaye71@yahoo.com](mailto:yonastesfaye71@yahoo.com)

**Thank you in advance!**

Novel Coronavirus (SAR-COV-19): What we know about it?

Part I: Sociodemographic characteristics

Q1. What is your gender?

- Male
- Female

Q2. What is your age (full years) ____________________

Q3. What is the type of your profession?_________________________

Q4. What is the level of your educational status?

- Diploma
- Bachelor Degree
- Masters degree
- PhD and above

Q5. What is your professional engagement (more than one response is possible)

- Clinical
- Academics

Q6. Where is your area of residence?

- Urban
- Rural

**Part II: Source of information about novel Coronavirus (SAR-COV-19) outbreak**

Q7. Have you heard of Novel Coronavirus?

- Yes (1)
- No (2)

Q8. How do you rate the sources of information you are using about the Novel Coronavirus (SAR-COV-19) outbreak?

Instruction: on a scale of 1 "least used sources" to 4 "most used sources", rate your response by ticking on the response for each question.

|  | Least used (1) | Sometimes (2) | More often (3) | Most used (4) |
| --- | --- | --- | --- | --- |
| Printed material |  |  |  |  |
| Social media (Facebook, Twitter, Whatsapp, YouTube, Instagram) |  |  |  |  |
| Official government websites (MOH, DHA, DOH, WHO, CDC) |  |  |  |  |
| Radio |  |  |  |  |
| Television |  |  |  |  |
| Family member, colleague or friend (4) |  |  |  |  |

**Part III: Question related precautionary behavioral practice towards novel coronavirus (SAR-COV-19)**

Q9. How do you rate your precautionary behavioral practice ( for question number xx to xxx) toward novel coronavirus (SAR-COV-19) outbreak?

**Instruction**: on a scale of 1 "least used" to 5 "all the time", how do you rate your precautionary behavioral practice ( for question number xx to xxx) toward novel coronavirus (SAR-COV-19) outbreak?

| Questions | Least frequently (1) | Sometimes (2) | More often (3) | Most used (4) | All the time (5) |
| --- | --- | --- | --- | --- | --- |
| 9.1. How often do you avoid people sneezing or coughing? |  |  |  |  |  |
| 9.2. How often do you avoid large gathering? |  |  |  |  |  |
| 9.3. How often do you avoid touching face, mouth, eye and nose? |  |  |  |  |  |
| 9.4. How often do you wash hands frequently? |  |  |  |  |  |
| 9.5. How often do you avoid public places/public transportation? |  |  |  |  |  |
| 9.6. How often do you avoid travel to affected areas? |  |  |  |  |  |
| 9.7. How often do you use alcohol based disinfectant? |  |  |  |  |  |
| 9.8. How often do you avoid spitting on the ground? |  |  |  |  |  |
| 9.9. How often are you wearing a mask? |  |  |  |  |  |
| 9.10. How often are you wearing gloves? |  |  |  |  |  |

Part IV: Question to assess health professionals risk perception, disease severity, vulnerability, and self-efficacy

Please rate your response for the following question

| Questions | No (1) | Minimal (2) | Moderate (3) | High (4) | Very high (5) |
| --- | --- | --- | --- | --- | --- |
| 10. How do you rate perceive risk of getting infected by COVID-19? |  |  |  |  |  |
| 11. How do you rate perceive risk of having serious illness by COVID-19? |  |  |  |  |  |
| 12. How do you rate perceive risk of death by COVID-19? |  |  |  |  |  |
| 13. How do you rate perceived effect that Corona virus pandemic poses difficulty in your life? |  |  |  |  |  |
| 14. How do you rate that Ethiopian are to contract the virus? |  |  |  |  |  |
| 15. How do you rate that that people in your present location are to contract the Coronavirus? |  |  |  |  |  |
| 16. How do you rate your worry that your family members or friend might be infected by Corona Virus? |  |  |  |  |  |
| 17. How do you rate the severity of COVID-19? |  |  |  |  |  |
| 18. How do you rate the severity of HIV/AIDS infection? |  |  |  |  |  |
| 19. How do you rate the severity of common cold? |  |  |  |  |  |
| 20. How do you rate the severity of malaria infection? |  |  |  |  |  |
| 21. How do you rate the severity of tuberculosis infection? |  |  |  |  |  |
| 22. How do you rate your ability to avoid infection with COVID-19? |  |  |  |  |  |
| 23. How do you rate your vulnerability of COVID-19? |  |  |  |  |  |
| 24. How do you rate your vulnerability of HIV/AIDS? |  |  |  |  |  |
| 25. How do you rate your vulnerability of common cold? |  |  |  |  |  |
| 26. How do you rate your vulnerability of malaria? |  |  |  |  |  |
| 27. How do you rate perceived severity of tuberculosis? |  |  |  |  |  |
| 28. How do you rate perceived ability to avoid infection with COVID-19? |  |  |  |  |  |
